# Digital Quadrupole Isolation and Electron Capture Dissociation on an Extended Mass Range Q-TOF Provides Sequence and Structure Information on Proteins and Protein Complexes

**DOI:** 10.1021/jasms.3c00184

**Published:** 2023-07-18

**Authors:** Carter Lantz, Robert Schrader, Joseph Meeuwsen, Jared Shaw, Noah T. Goldberg, Shane Tichy, Joe Beckman, David H. Russell

**Affiliations:** † Department of Chemistry, Texas A&M University, College Station, Texas 77843, United States; ‡ e-MSion, a part of Agilent, 2121 NE Jack London St, Ste 140, Corvallis, Oregon 97330, United States; § Agilent Technologies, 5301 Stevens Creek Blvd, Santa Clara, California 95051, United States

**Keywords:** native mass spectrometry, digital quadrupole, electron capture dissociation, charge reduction, protein structure

## Abstract

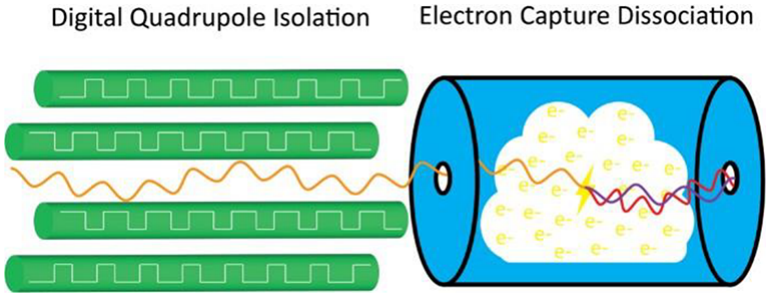

Electron capture dissociation (ECD) is now a well-established method for sequencing peptides and performing top-down analysis on proteins of less than 30 kDa, and there is growing interest in using this approach for studies of larger proteins and protein complexes. Although much progress on ECD has been made over the past few decades, establishing methods for obtaining informative spectra still poses a significant challenge. Here we describe how digital quadrupole (DigiQ) ion isolation can be used for the mass selection of single charge states of proteins and protein complexes prior to undergoing ECD and/or charge reduction. First, we demonstrate that the DigiQ can isolate single charge states of monomeric proteins such as ubiquitin (8.6 kDa) and charge states of large protein complexes such as pyruvate kinase (234 kDa) using a hybrid quadrupole-TOF-MS (Agilent extended *m*/*z* range 6545XT). Next, we demonstrate that fragment ions resulting from ECD can be utilized to provide information about the sequence and structure of the cytochrome c/heme complex and the ubiquitin monomer. Lastly, an especially interesting result for DigiQ isolation and electron capture (EC) was noted; namely, the 16+ charge state of the streptavidin/biotin complex reveals different electron capture patterns for the biotinylated proteoforms of streptavidin. This result is consistent with previous reports that apo streptavidin exists in multiple conformations and that biotin binding shifts the conformational dynamics of the complex (Quintyn, R. Chem. Biol.2015, 22 ( (5), 5), 583−59225937312 10.1016/j.chembiol.2015.03.019).

## Introduction

Native mass spectrometry (MS) involves the ionization and subsequent mass measurement of intact proteins and protein complexes under conditions that preserve elements of the solution-phase structure in the gas-phase ions.^1,2^ This technique is routinely used to determine the molecular weight of intact protein ions and protein complexes such as the GroEL 14mer^3,4^ and can even provide mass information on 18 MDa virus assemblies.^5^ In addition, native MS measurements can provide information on the identity of modifications that are covalently or noncovalently attached to the protein.^6−8^ Native MS has grown in popularity in the realm of structural biology because of the dynamic range and resolution that the technique provides. For example, Yang et al. measured 59 different proteoforms of chicken ovalbumin including 45 different glycan structures with a single mass measurement.^9^ In another study, multiple copper binding events could be observed for the ABC transporter MsbA.^10^ These two recent examples illustrate the dynamic range and resolution that native MS can provide and have spurred the development of techniques that can be utilized to further interrogate these large biopolymers.

Isolation of the charge states and proteoforms of native proteins is an integral part of protein mass spectrometry analysis. Isolation is commonly utilized to interrogate specific ion populations in a mass spectrum for a more precise analysis of their sequence or structure. For the sinusoidally driven quadrupole mass filter, typical on commercial quadrupole/time-of-flight instruments, the mass range is limited by the maximum RF and DC voltages. Higher mass range can be achieved at lower voltages by reducing the RF drive frequency.^11,12^ Alternatively, digitally driven quadrupole mass filters operate by adjusting the drive frequency while maintaining a constant RF voltage which allows for selection of high *m*/*z* values at low voltage.^13−15^ By changing the duty cycle (the percentage of the waveform at +V_RF_ and –V_RF_) (see the Supporting Information, Figure S1A), the stability diagram is manipulated such that only a small range of Mathieu *q* values are stable at *a* = 0^16^ (Figure S1B). Our results in this work show that digital quadrupole (DigiQ) isolation can be adapted to a commercial Agilent 6545XT Q-TOF instrument and efficiently isolate charge states of small monomeric proteins and larger (234 kDa) protein complexes.

Electron capture dissociation (ECD) is a native top-down mass spectrometry (TD-MS) technique that utilizes the capture of electrons to generate fragment ions that can be utilized to sequence proteins. The activation provided by the electron induces rearrangement of bonds allowing the cleavage of the N–Cα bond and the release of *c*- and *z*-fragment ions.^17^ ECD has been reported to dissociate peptide bonds without significantly disrupting interactions between weakly bound modifications and proteins as well as intramolecular interactions between protein residues. Because of this fact, fragment ions resulting from ECD have been utilized to locate weakly bound post-translational modifications,^7,18^ metal ions,^19,20^ and electrostatically bound compounds^7,19^ along a protein sequence. Furthermore, fragment ions resulting from ECD have been utilized to probe well-ordered regions of monomeric proteins,^21−23^ and solvent-exposed regions of protein complexes.^24,25^ Our results in this work show that ECD after DigiQ isolation can release fragment ions that provide sequence, modification, and structure information on protein ions. Additionally, we demonstrate that electron capture by protein complexes can provide information about conformational shifts upon ligand binding to the complex. These promising new developments demonstrate that the interrogation of native protein ions with DigiQ isolation and ECD can be a useful tool for structural biology.

## Methods

Proteins such as ubiquitin, cytochrome c, alcohol dehydrogenase, and pyruvate kinase were purchased from Sigma-Aldrich. Protein solutions were buffer exchanged into 20–200 mM ammonium acetate with Bio-Spin 6 SEC columns (BioRad) and diluted to 2.5–17 μM. Streptavidin was sprayed at 40−53 μM, and biotin was titrated into the solution until binding could be observed. The solutions were inserted into either a 250 μL Hamilton syringe or custom-pulled nanospray capillaries (Sutter Instruments, BF150-86-10) before electrospraying the solution.

Solutions were sprayed on an Agilent 6545XT (Agilent Technologies, Santa Clara, CA) and sprayed with either a Dual AJS ESI source using a source voltage value of 4 kV or a nanospray source using source voltage values ranging from 1.2 to 2.0 kV. The digital quadrupole was adapted to the instrument in place of the sinusoidal quadrupole for charge state isolation. The digital quadrupole was operated by using waveform generators provided by Gordon Anderson Custom Electronics. Full scans were collected by using a duty cycle of 50.0/50.0. Ion populations were isolated by shifting the duty cycle from 60.75/39.25 to 61.2/38.8 and varying the frequency based off a *q*-value of 0.59 and using a voltage of 50–100 V_0-p_. FWHM values were obtained by fitting lines to the data using *x* as *m*/*z* and *y* as intensity. The *m*/*z* value at 50% peak height was then calculated from the fitted lines.

Electron-capture dissociation was performed with a prototype ExD cell (e-MSion, Corvallis, OR). The FB parameter and the L4 parameter on the ECD cell were varied to generate charge reduction and fragment ions from the intact precursor. ECD spectra were deconvoluted with the eTHRASH algorithm^26^ using MASH Native^27^ with 5–10 signal-to-noise ratio and a min fit parameter of 80–85% and matched with ClipsMS.^28^ For cytochrome c, 20 ppm error was allowed for identification of *c*- and *z*-fragment ions, a heme group and a hydrogen atom were added as unlocalized modifications, and an acetylation modification was added as an N-terminal modification. For ubiquitin, 20 ppm error was allowed for identification of *c*- and *z*-fragment ions, and the addition of the hydrogen atom was added as an unlocalized modification. All fragment types were assigned with ExDViewer v4.3.40. Intact mass deconvolution was performed with either Protein Metrics or UniDec.^29^ Structure analysis was performed with PyMOL version 2.5.4 using PDB codes 1HRC for cytochrome c and 1UBQ for ubiquitin. For the EC experiments with lower concentrations of biotin, 46 μM biotin was inserted into 53 μM streptavidin and buffer exchanged into 200 mM ammonium acetate before electrospraying the solution. Zinc binding was endogenous to one of the samples. To obtain all biotin-bound states, 200 μM biotin was inserted into 40 μM streptavidin. After biotin was inserted, the solution was buffer exchanged into 200 mM ammonium acetate to remove excess salt and biotin. Then the apo streptavidin was inserted into the solution at an equimolar ratio before electrospraying. The solutions were either kept on ice or stored at 4 °C before electrospraying on the instrument. Three or four replicates were collected for each solution, and error bars correspond to the standard deviation of the replicates.

## Results and Discussion

### Digital Quadrupole Isolation of Native Protein Charge States

**Figure 1:**
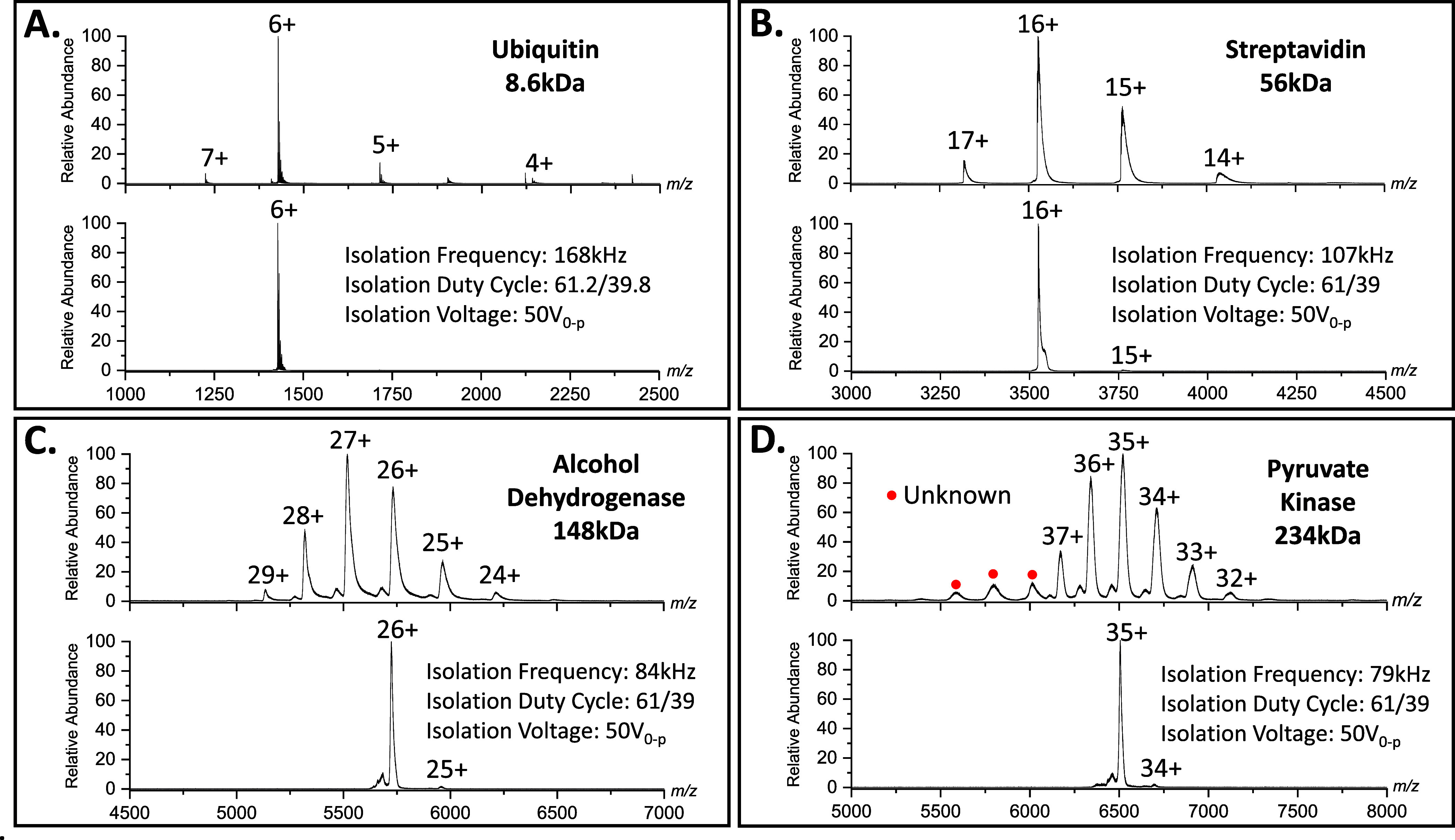
Spectra of (A) ubiquitin, (B) streptavidin, (C) alcohol dehydrogenase, and (D) pyruvate kinase collected at a 50.0/50.0 duty cycle and a spectrum corresponding to the isolation of a charge state with the DigiQ. The isolation spectra reveal that the digital quadrupole can readily isolate charge states of small protein monomers and large protein complexes.

DigiQ mass filters have previously been found to routinely isolate charge states of native protein ions on an in-house built instrument.^14,15^ To test the isolation capability of the DigiQ on a commercial instrument, charge states of proteins with various molecular weights and *m*/*z* values (Figure S2) were isolated on an Agilent 6545XT Q-TOF instrument. A native solution of ubiquitin (8.6 kDa) was electrosprayed, and the 6+ charge state of the protein (1428 *m*/*z*) was isolated with the DigiQ at a duty cycle of 61.2/38.8 and 50 V_0-p_ (Figure 1A). The waveform applied to the quadrupole allowed the 6+ charge state to pass through the filter and excluded all other charge states that were present in the MS1 spectrum. In addition, a native solution of cytochrome *c* (12.4 kDa) was electrosprayed, and the 7+ charge state (1766 *m*/*z*) of the protein was isolated with a duty cycle of 61.2/38.8 and 50 V_0-p_ (Figure S3). The waveform applied to the quadrupole allowed the 7+ charge state to pass through the filter and excluded all other charge states that were present in the MS1 spectrum. These examples show that the DigiQ can routinely isolate charge states of native monomeric proteins on a commercial instrument.

The DigiQ can also be utilized to isolate charge states of native protein complexes. Performing isolation of ions with high *m*/*z* values can be difficult with a traditional sinusoidal quadrupole because the voltage required to isolate those ions becomes prohibitive. Charge states of protein complexes often appear at these high *m*/*z* values; therefore, isolation of those peaks cannot be performed. However, our results indicate that the DigiQ can routinely isolate charge states at high *m*/*z* values without the need for high voltage values. To demonstrate this, charge states of various protein complexes that exist at high *m*/*z* values were isolated with the DigiQ on the Agilent 6545XT Q-TOF. The native streptavidin tetramer (56 kDa) was electrosprayed, and the 16+ charge state (3524 *m*/*z*) was isolated with a duty cycle of 61.0/39.0 and 50 V_0-p_ (Figure 1B). The resulting spectrum revealed the presence of the 16+ charge state and that the other charge states were largely excluded from the spectrum, indicating the DigiQ was able to isolate the charge state at that duty cycle. The alcohol dehydrogenase (ADH) tetramer (148 kDa) was electrosprayed, and the 26+ charge state (5732 *m*/*z*) was isolated with a duty cycle of 61.0/39.0 and 50 V_0-p_ (Figure 1C). The resulting spectrum revealed the 26+ charge state as well as the 26+ charge state of a lower abundance proteoform. The other charge states are largely absent from the spectrum, indicating the DigiQ isolation of that charge state was successful at that duty cycle. Pyruvate kinase (234 kDa) was also electrosprayed, and the 35+ charge state (6521 *m*/*z*) of the protein was isolated with a duty cycle of 61.0/39.0 and 50 V_0-p_ (Figure 1D). The spectrum revealed the 35+ charge state as well as the 35+ charge state of a lower abundance proteoform. The other charge states are largely absent from the spectrum and the 150 kDa unknown protein is absent from the spectrum indicating the DigiQ can successfully isolate charge states >6500 *m*/*z*. Notice in the isolation spectra for these protein complexes that there are low abundance peaks corresponding to lower charge states in each spectrum. It is possible that those signals are present because of charge stripping after isolation with the DigiQ. Protein ions that are stripped of a positive ion would then manifest as a lower charge state. This is further supported by the fact that the isolated charge states contain fewer adducts compared to the corresponding charge states in the MS1 spectrum. Nevertheless, these examples suggest that DigiQ isolation can routinely isolate charge states of native protein complexes and can be used for further interrogation of those complexes.

The resolution of the digital quadrupole was determined at various duty cycles by varying the frequency with which an ion was isolated. First, the resolution for Agilent tune mix ions was measured. At an *m*/*z* value 322, the full width at half-maximum (FWHM) value was 2 *m*/*z* at duty cycle 61.0/39.0 corresponding to a resolution of 156 and 0.5 *m*/*z* at duty cycle 61.1/38.9 corresponding to a resolution of 609 (Figure S4A). At the *m*/*z* value 1221, the FWHM value was 13 *m*/*z* at duty cycle 61.0/39.0 corresponding to a resolution of 91 and 7 *m*/*z* at duty cycle 61.1/38.9 corresponding to a resolution of 187 (Figure S4B). At the *m*/*z* value of 2721, the FWHM value was 33 *m*/*z* at duty cycle 61.0/39.0 corresponding to a resolution of 82 and 14 *m*/*z* at duty cycle 61.1/38.9 corresponding to a resolution of 189 (Figure S4C). Next the resolution of the digital quadrupole was determined for the 6+ charge state of ubiquitin and the 26+ charge state of ADH. For the 6+ charge state at *m*/*z* value 1428, the FWHM value was 18 *m*/*z* at duty cycle 61.0/39.0 corresponding to a resolution of 80 and 9 *m*/*z* at duty cycle 61.1/38.9 corresponding to a resolution of 163 (Figure S4D). For the 26+ charge state of ADH at a *m*/*z* value of 5714, the isolation window was determined to be 104 *m*/*z* at duty cycle 61.0/39.0 which corresponds to a resolution of 55 (Figure S4E). Not enough signal could be averaged at a 61.1/38.9 duty cycle, so ADH data for that duty cycle was not recorded. This data reveals that ∼2x resolution can be afforded by the 61.1/38.9 duty cycle, but the signal decreased by ∼2x. However, the resolution at either duty cycle is sufficient for isolation of protein charge states for subsequent analysis with electron capture and electron capture dissociation.

### Electron Capture Dissociation (ECD) of Native Protein Ions

**Figure 2:**
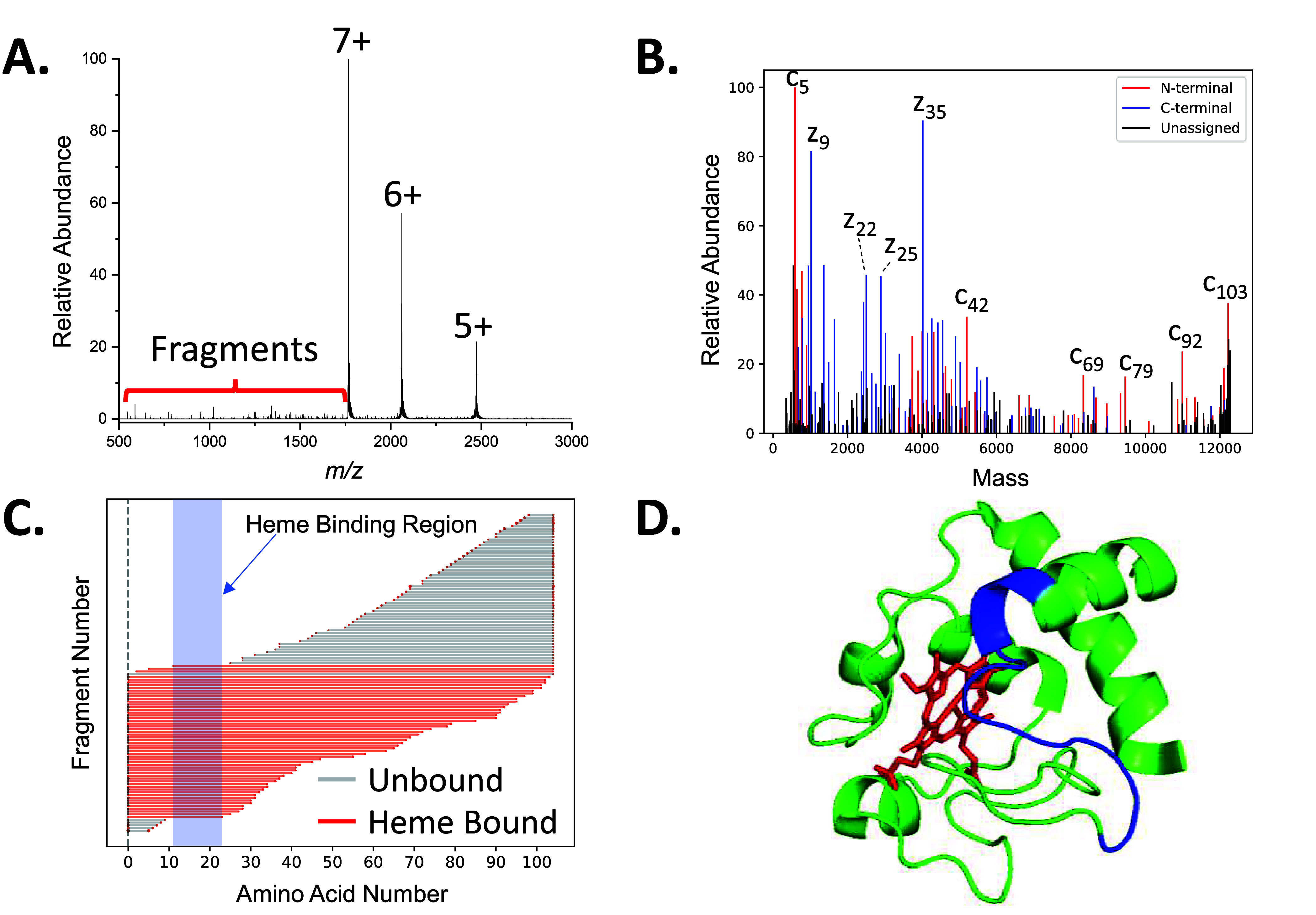
A.) An ECD spectrum of the 7+ charge state of native cytochrome *c* with B.) the deconvoluted spectrum revealing multiple *c*- and *z*-fragment ions are present in the spectrum. C.) The fragment ions localized an acetylation modification on the N-terminus and the heme binding site at residues 12–23. D.) The crystal structure reveals that residues 12–23 (blue) interact with the heme group (red).

Previous studies have found that ECD of protein complexes can release fragment ions that reveal sequence information and retain covalent modifications.^7,19^ Our results suggest that ECD with a prototype ExD cell can provide similar information about protein ligand complexes. The 7+ charge state of native cytochrome c with a heme molecule bound was isolated with a 61.2/38.8 duty cycle on the DigiQ and subsequent ECD was performed on the protein complex (Figure 2A). The spectrum revealed multiple charge reduced precursor signals and numerous low-abundance fragment ions. Deconvolution of the fragment ions in the spectrum revealed multiple mass spectral signals corresponding to *c*- and *z-*fragment ions which added up to a sequence coverage (percent of inter-residue cleavage sites) of 74% (Figure 2B). Analysis of all fragment ions in the spectrum added up to a sequence coverage of 96% (Figure S5). Other studies have reported lower sequence coverage values for cytochrome c with gentle conditions;^22^ however, the high signal-to-noise for our spectra acquired using DigiQ ion selection increases the dynamic range for ion detection. Mapping the fragment ions to the sequence reveals that the acetylation site is on the N-terminus and the heme molecule binds and interacts with residues 12–23 (Figure 2C). No *c*- or *z*-fragment ions are observed between residues 12–23 presumably because the heme group stabilizes that region of the protein. The crystal structure of the protein confirms that those residues hold the heme group in the binding pocket of the protein (Figure 2D). This example demonstrates that the ECD of native protein ions with the prototype cell can provide sequence information and the location of covalent modifications along the sequence of the protein.

Previous studies have also found that fragment ions from ECD are sensitive to the structural characteristics of protein ions.^30,31^ To demonstrate this capability with the prototype ExD cell, the 6+ charge state of ubiquitin was isolated with the DigiQ and subsequent ECD was performed on the isolated charge state (Figure S6A). The spectrum revealed charge reduced precursor signals as well as low abundance fragment ions. Deconvolution of the spectrum revealed multiple *c*- and *z*-fragment ions corresponding to the protein sequence, which added up to a sequence coverage of 85% (Figure S6B). Analysis of all fragment ions in the spectrum added up to a sequence coverage of 100% (Figure S6C). The fragmentation map shows extensive sequence coverage at the N- and C-termini; however, only low abundance ions are detected for the region between residues 34–48 which only account for 0.43% of the total fragment ion current (Figure S6D). The crystal structure of ubiquitin reveals that this region corresponds to part of a β sheet structure of the protein monomer (Figure S6E). This finding lines up well with those from other ECD studies of ubiquitin. In one study, ECD of activated ubiquitin revealed that region III of ubiquitin (residues 36–50) had the highest gas-phase stability.^23^ Zhang et al. reported that this region has a low B-factor indicating the region is well ordered.^21^ It is possible that the intramolecular interactions between the residues of the β sheet structure hold the protein together even if the covalent bonds of the region are cleaved. We find that *c*- and *z*-fragment ions were shown to stem from either terminus, but the center of the protein sequence where the β sheet interaction is located does not fragment as readily. This example demonstrates that ECD of native protein ions with the ExD prototype cell can provide structure information about native protein ions.

### Conformational Analysis of Native Protein/Ligand Complexes with Electron Capture (EC)

**Figure 3:**
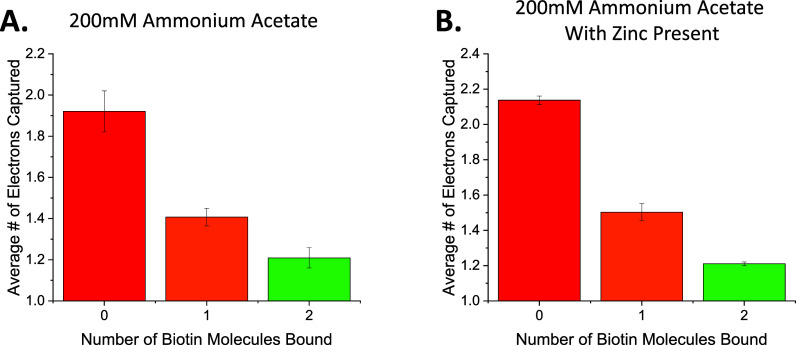
Average number of electrons captured for apo streptavidin as well as streptavidin bound with 1 biotin molecule and streptavidin bound with 2 biotin molecules in A.) 200 mM ammonium acetate and B.) 200 mM ammonium acetate with Zn^2+^ present. The complexes that bind biotin capture fewer electrons, indicating that biotin binding shifts the conformational dynamics of the complex.

It is well established that performing ECD produces charge reduced precursor signals in addition to fragment ions; however, these charge reduced precursor signals are largely ignored, as fragmentation tends to be the preferred result of ECD analysis. Although, it is possible that electron capture (EC) can be utilized as an indicator for protein conformation shifts. To test this hypothesis, EC was performed on a solution containing the apo streptavidin tetramer (56 kDa) as well as proteoforms of the streptavidin/biotin complex. The 16+ charge state of apo streptavidin and multiple streptavidin/biotin complexes were isolated with a duty cycle of 61.0/39.0, and EC was performed revealing multiple signals corresponding to charge reduced precursors of the proteoforms (Figure S7A). Analysis of multiple replicates revealed that apo streptavidin captured an average of 1.92 electrons, while the streptavidin complex binding 1 molecule of biotin captured an average of 1.41 electrons and the streptavidin complex binding 2 molecules of biotin captured an average of 1.21 electrons (Figure 3A). The fact that less electrons are captured for biotin-bound proteoforms indicates that biotin shifts the conformational dynamics of the tetramer relative to the unbound complex. EC was also performed on the biotin/streptavidin complex in a solution containing Zn^2+^ (Figure S7B). Analysis of the proteoforms that did not bind Zn^2+^ ions indicated that apo streptavidin captured an average of 2.14 electrons, while the streptavidin complex binding 1 biotin molecule captured an average of 1.50 electrons and the streptavidin complex binding 2 biotin molecules captured an average of 1.21 electrons. This data further suggests that biotin binding shifts the structure of the streptavidin tetramer. Previous data indicate that the unbound tetramer exists in multiple conformations.^32^ Following binding of one biotin molecule, there is a shift in quaternary structure of the complex.^33^ In addition it is known that a flexible loop modulates biotin binding to the tetramer.^34,35^ With knowledge of the shifting dynamics of the complex upon biotin binding, it may be concluded that the difference in EC pattern may be indicative of a changing quaternary structure and changes in loop dynamics when biotin binds the complex. While this interpretation of the EC results is rather speculative, conformational analysis of the products of ECD using alternative approaches, viz. variable-temperature ESI^6,36^ and ion mobility spectrometry,^37,38^ is underway.

**Figure 4:**
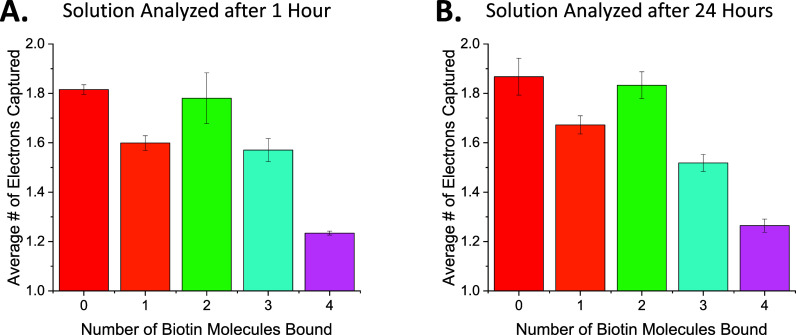
Average number of electrons captured for apo streptavidin and all streptavidin/biotin complexes after A.) 1 h of incubation and B.) 24 h of incubation. The data provide evidence that each biotin binding event shifts the dynamics of the complex.

EC was also performed on apo streptavidin and streptavidin–biotin proteoforms to probe the dynamics of the complex upon biotin binding. Solutions containing all proteoforms were prepared, and the 16+ charge state was isolated with the DigiQ at a duty cycle of 60.75/39.25. This wider window allowed for all biotin states of the 16+ charge state to pass through the filter but did not allow other charge states to pass through the filter (Figure S8). After 1 h of incubation, EC of the 16+ charge state was performed which revealed charge reduced precursor peaks for all isolated proteoforms of the complex (Figure S9A). Apo streptavidin captured an average of 1.81 electrons, the 1 biotin bound proteoform captured an average of 1.60 electrons, the 2 biotin bound proteoform captured an average of 1.78 electrons, the 3 biotin bound proteoform captured an average of 1.57 electrons, and the 4 biotin bound proteoform captured an average of 1.23 electrons (Figure 4A). The data suggest that the dynamics of the streptavidin tetramer is altered upon biotin binding. The fact that streptavidin with 4 biotin molecules bound captures fewer electrons than apo streptavidin suggests that biotin compacts the structure of the protein. Previous ion mobility data shows that 4 biotin bound streptavidin has a smaller collision cross section (CCS) than apo streptavidin which is consistent with formation of more compact conformers.^32^ The proteoform with 1 biotin molecule bound and the proteoform with 3 biotin molecules bound capture a similar number of electrons, indicating that their conformational dynamics may be similar. Interestingly, the proteoform with 2 biotin molecules bound captures more electrons than the proteoforms with 1 biotin molecule bound which differs from the data in Figure 3. It is possible that the structures in this solution that bind 2 biotin molecules adopt a conformation that cannot bind more molecules of biotin. The data suggest that EC of protein complexes is sensitive to their structure and can provide information on the dynamics of the complex. This solution was also analyzed after 24 h of incubation at 4 °C. DigiQ isolation and EC were performed on the 16+ charge state containing all proteoforms which revealed charge reduced precursors for all complexes (Figures S9B). Apo streptavidin captured an average of 1.87 electrons, the 1 biotin bound proteoform captured an average of 1.67 electrons, the 2 biotin bound proteoform captured an average of 1.83 electrons, the 3 biotin bound proteoform captured an average of 1.52 electrons, and the 4 biotin bound form captured an average of 1.26 electrons (Figure 4B). This data suggests that apo streptavidin still exists in a more extended state compared to the proteoform with 4 biotin molecules bound, although the 1 biotin bound and 3 biotin bound proteoforms of the protein captured a different number of electrons from one another which contrasts with those same complexes at 1 h (Figure 4A). It is possible that incubation for 24 h allows the structure of the streptavidin tetramers to equilibrate. Lastly, the proteoform of streptavidin with 2 biotin molecules bound showed very similar behavior at 24 h compared to the behavior at 1 h. As stated previously, it is possible that the structures that bind 2 biotin molecules adopt a conformation that cannot bind more biotin molecules in this solution. This data further indicate that EC can probe the dynamics of protein/ligand complexes and can provide information on how the ligands shift their structure.

## Conclusions

Here we extend the capability of an Agilent Q-TOF instrument to analyze native proteins and protein complexes with the incorporation of DigiQ isolation and ECD. DigiQ isolation was found to isolate charge states of small native protein monomers and larger protein complexes with charge states up to ∼6500 *m*/*z*, extending the isolation capability of the quadrupole, which is traditionally limited to 4000 *m*/*z*. DigiQ isolation with subsequent ECD of the cytochrome c/heme complexes reveals the release of *c*- and *z*-fragment ions that can be utilized to localize the acetylated residue on the N-terminus and the heme binding region between residues 12–23. In addition, it was found that ECD of the ubiquitin monomer released fragment ions that are sensitive to the structure of the protein, specifically the location of an ordered β sheet region between residues 34 and 48. Furthermore, isolation and EC of the 16+ charge state of streptavidin revealed different charge reduction patterns for apo streptavidin and its corresponding biotinylated proteoforms, which suggests that biotin shifts the conformational dynamics of the streptavidin tetramer. The data presented here demonstrate that DigiQ isolation and ECD can be utilized to provide information on native proteins and protein complexes and may enhance our understanding of protein dynamics and structural biology.

## Supporting Information Available

Representation of the digital quadrupole, mass information on the proteins analyzed in this study, isolation of cytochrome c, digital quadrupole resolution plots, ECD analysis of ubiquitin, and electron capture analysis of streptavidin (PDF)
