# Supplementary material for: Digital Quadrupole Isolation and Electron Capture Dissociation on an Extended Mass Range Q-TOF Provides Sequence and Structure Information on Proteins and Protein Complexes
Source: J Am Soc Mass Spectrom. 2023 Jul 18;34(8):1753–60. doi: 10.1021/jasms.3c00184 (PMC10496594; doi:10.1021/jasms.3c00184)
Supplement: Supplementary file 1 — js3c00184_si_001.pdf [file js3c00184_si_001.pdf]

Supporting information

Digital Quadrupole Isolation and Electron Capture Dissociation on an Extended Mass Range Q-TOF Provides Sequence and Structure Information on Proteins and Protein Complexes

Authors:

Carter Lantz,[1] Robert Schrader,[1] Joseph Meeuwsen,[2] Jared Shaw,[2] Noah T. Goldberg,[3] Shane Tichy,[3] Joe Beckman,[2] David H. Russell[1]*

[1]Department of Chemistry, Texas A&M University, College Station, TX 77843

[2]e-MSion, a part of Agilent, 2121 NE Jack London St, Ste 140, Corvallis, OR 97330

[3]Agilent Technologies, 5301 Stevens Creek Blvd, 95051, Santa Clara, CA, USA

*Corresponding author; Email: russell@chem.tamu.edu

Table of Contents

Figure	Page
Figure S1	S2
Figure S2	S2
Figure S3	S3
Figure S4	S4
Figure S5	S5
Figure S6	S6
Figure S7	S7
Figure S8	S8
Figure S9	S9

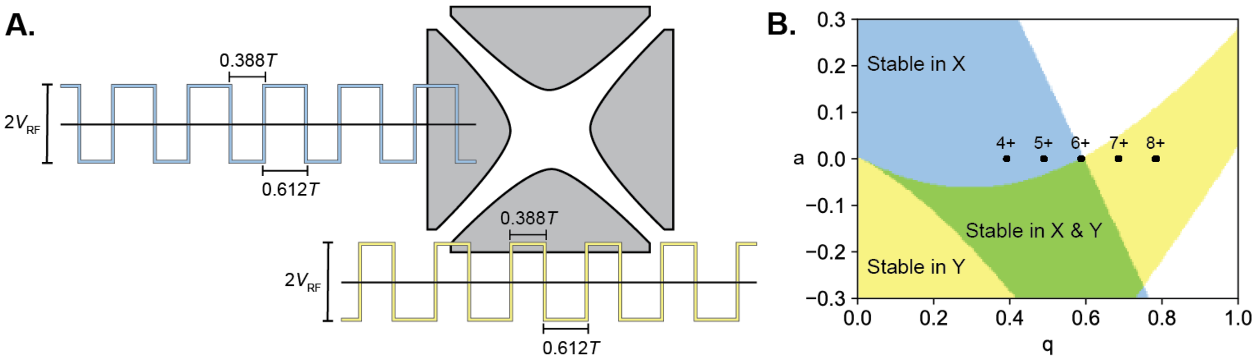

Figure S1: A.) A representation of how digital quadrupole isolation operates and B.) a corresponding Mathieu stability diagram showing how a digital quadruple isolates the 6+ charge state of ubiquitin. An asymmetric waveform is applied to the quadrupole rods which shifts the stability region of zone 1,1, allowing only the 6+ charge state of ubiquitin to be stable through the quadrupole.

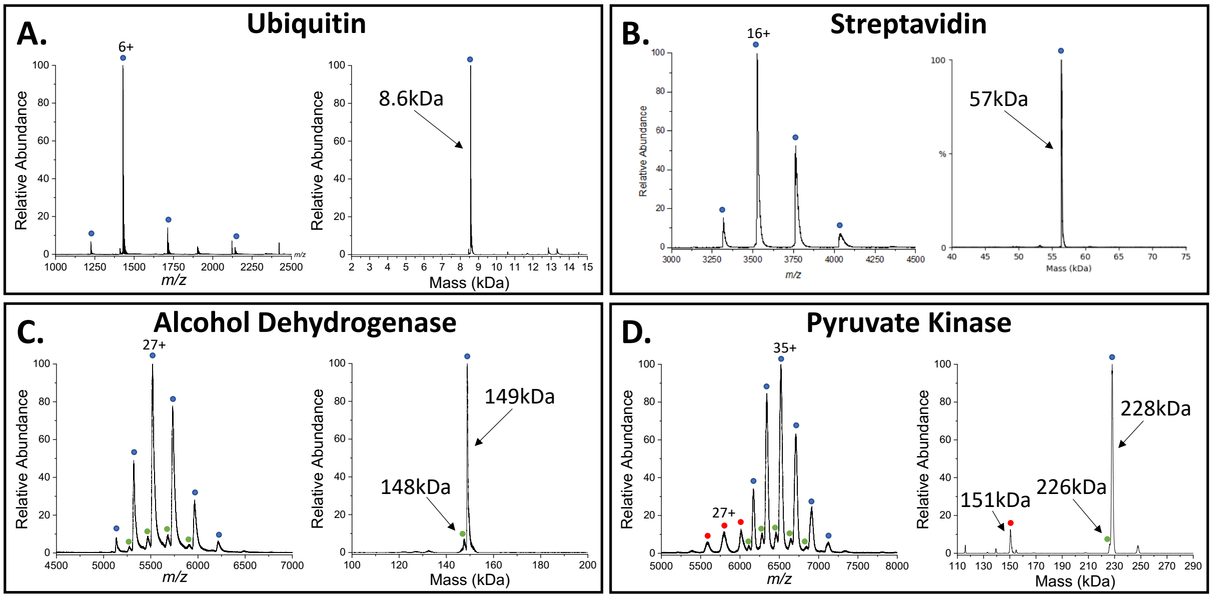

Figure S2: A Protein Metrics or UniDec deconvolution of the MS1 spectrum for A.) ubiquitin, B.) streptavidin, C.) alcohol dehydrogenase, and D.) pyruvate kinase. The blue dots indicate the base proteoform, the green dots indicate lower abundance proteoforms, and the red dots indicate unknown proteoforms.

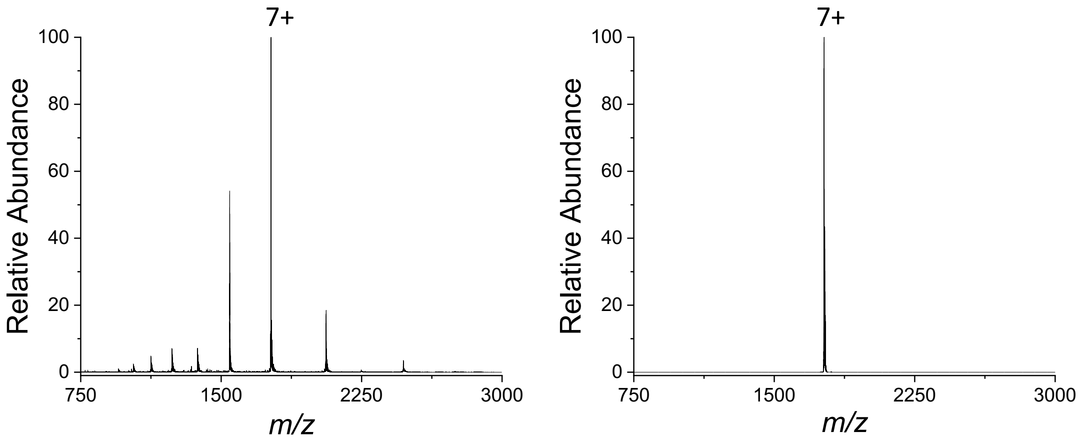

Figure S3: A native mass spectrum for cytochrome *c*/heme complex, and a spectrum of the 7+ charge state isolated with the DigiQ.

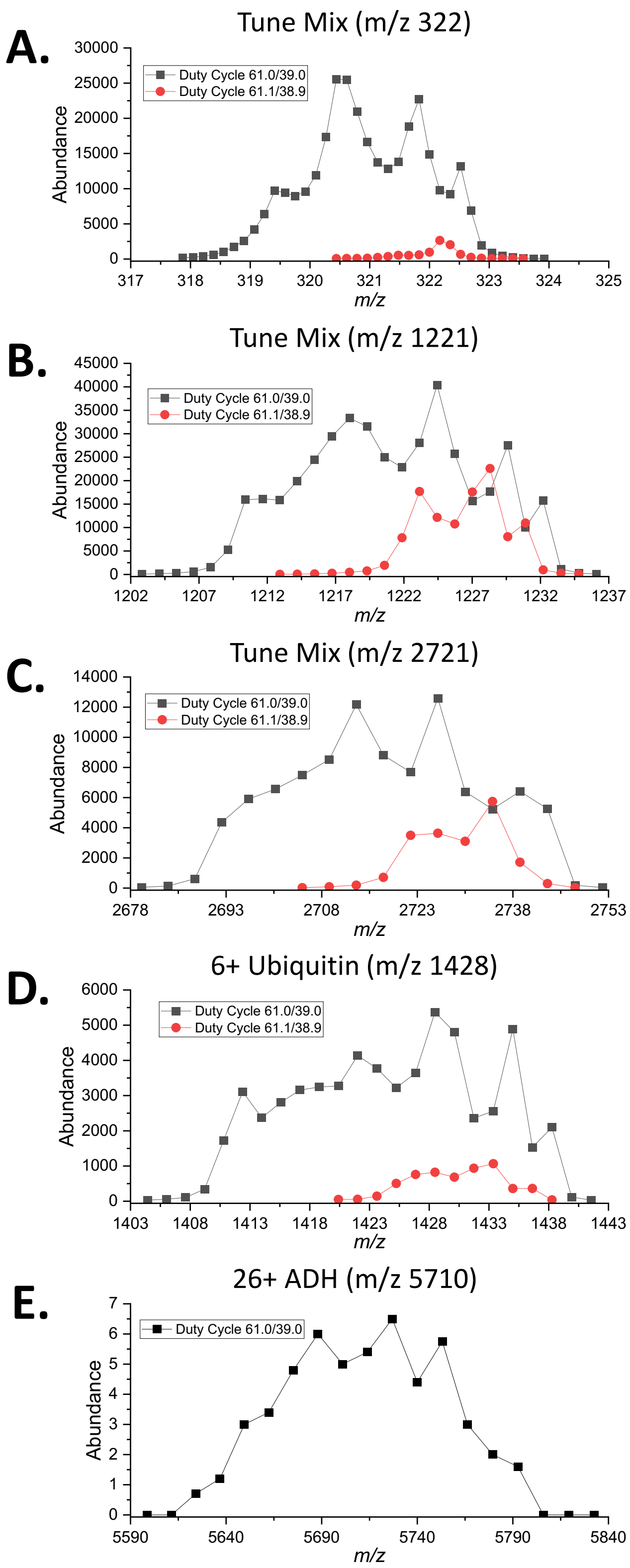

Figure S4: Plots determining the isolation windows at varying duty cycles for A.) tune mix peak 322, B.) tune mix peak 1221, C.) tune mix peak 2721, D.) The 6+ charge state of ubiquitin, and E.) the 26+ charge state of ADH.

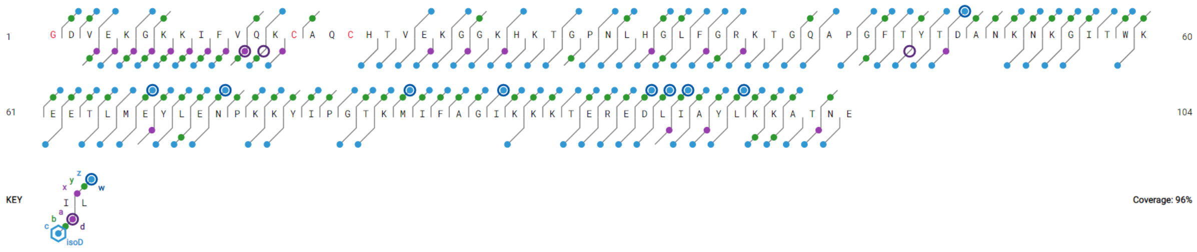

Figure S5: A fragment ion map representing all fragment ions present in the ECD spectrum of native cytochrome *c*.

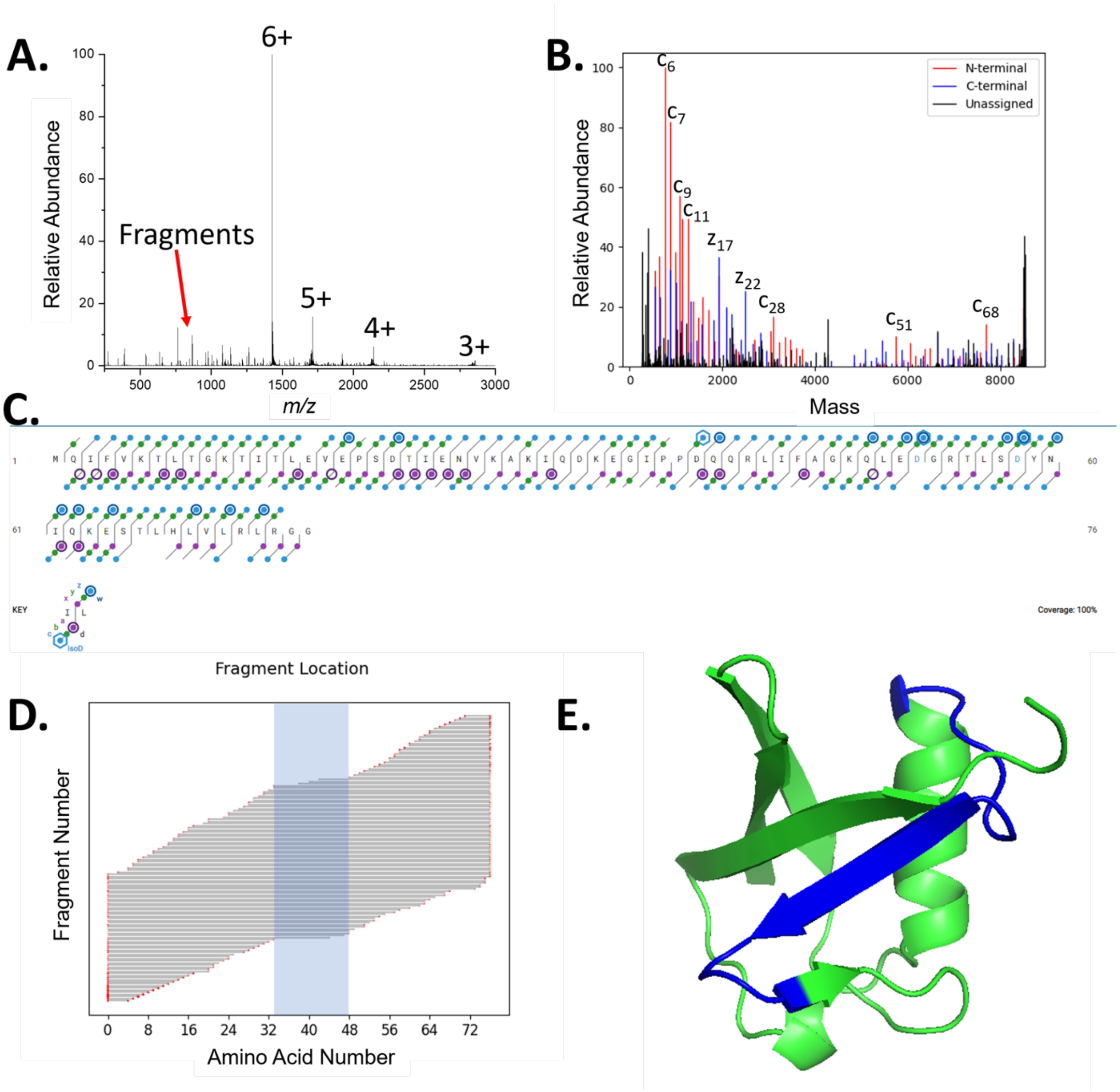

Figure S6: A.) An ECD spectrum of ubiquitin revealing charge reduction and multiple fragments of the protein with B.) the corresponding deconvoluted spectrum of the fragment ions, C.) a fragment ion map revealing all fragments present in the sample, and D.) a fragment ion map showing the *c*- and *z*-fragment ions identified in the spectrum. The fragment location map reveals a region of the sequence with very few fragment ions (blue region) which E.) corresponds to an ordered beta-sheet regions of the protein (blue region).

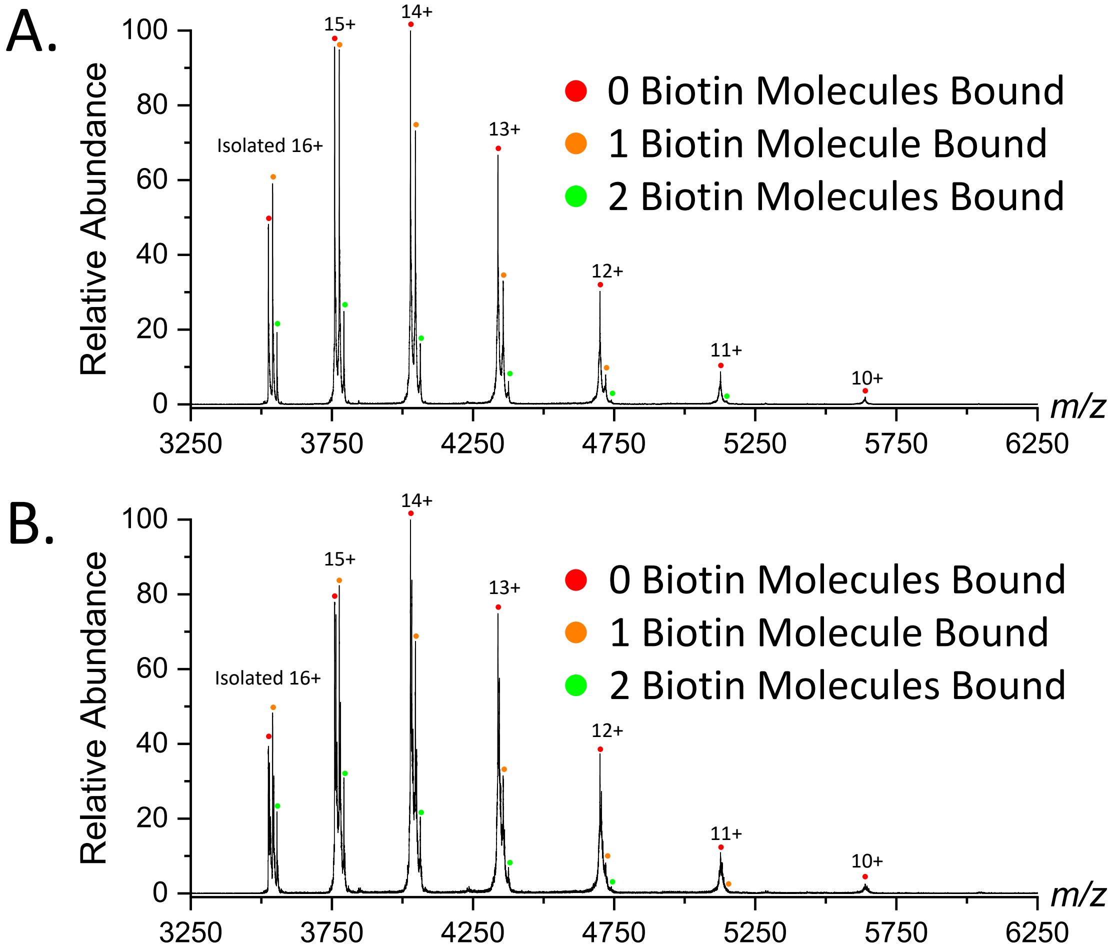

Figure S7: Mass spectra after DigiQ isolation and EC of the 16+ charge state of apo streptavidin, streptavidin with 1 biotin molecule bound, and streptavidin with 2 biotin molecules bound in A.) a solution of 200mM ammonium acetate and B.) a solution of 200mM ammonium acetate with zinc present. The proteoforms capture electrons differently as is shown by the difference in charge reduction pattern.

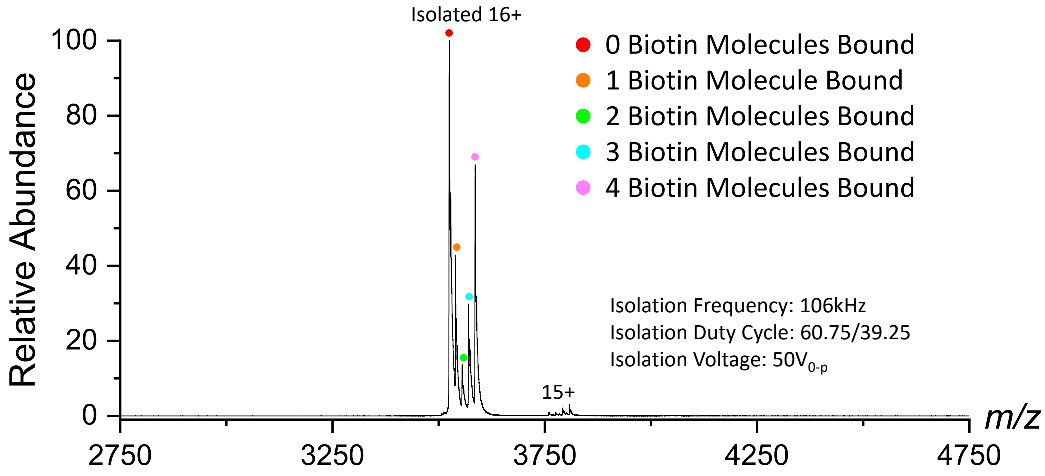

Figure S8: DigiQ isolation of the 16+ charge state of apo streptavidin and all streptavidin/biotin complexes at a duty cycle of 60.75/39.25. Notice that all complexes of the 16+ charge state are present and the other charge states are largely absent from the spectrum at this duty cycle.

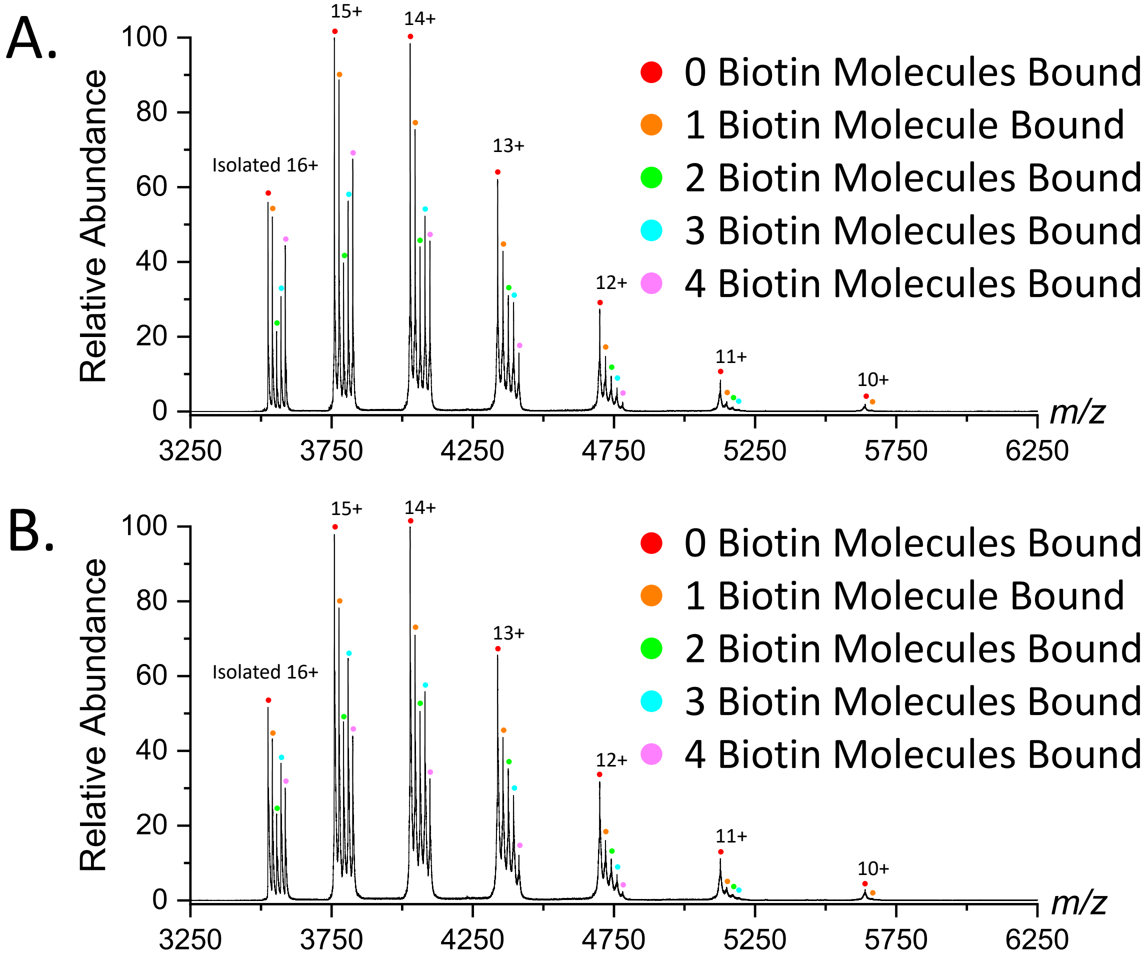

Figure S9: Spectra after DigiQ isolation and electron capture of the 16+ charge state of all biotin bound proteoforms of streptavidin analyzed after A.) 1 hour of incubation and B.) 24 hours of incubation. The proteoforms capture electrons differently as is shown by the difference in charge reduction pattern.
